# When and how does the number of children affect marital satisfaction? An international survey

**DOI:** 10.1371/journal.pone.0249516

**Published:** 2021-04-22

**Authors:** Marta Kowal, Agata Groyecka-Bernard, Marta Kochan-Wójcik, Piotr Sorokowski

**Affiliations:** Institute of Psychology, University of Wrocław, Wrocław, Poland; Hackensack Meridian Health, UNITED STATES

## Abstract

The present global study attempts to verify the links between marital satisfaction and the number of children as well as its moderators in an international sample. Data for the study was obtained from our published dataset and included 7178 married individuals from 33 countries and territories. We found that the number of children was a significant negative predictor of marital satisfaction; also sex, education, and religiosity were interacting with the number of children and marital satisfaction, while there were no interactions with economic status and individual level of individualistic values. The main contribution of the present research is extending our knowledge on the relationship between marital satisfaction and the number of children in several, non-Western countries and territories.

## Introduction

Paying attention to the lights and shadows of parenthood, researchers emphasize a multifaceted influence of becoming a parent on well-being [1–3], and more specifically, on marital satisfaction [4–6], especially while the number of children in a family grows [7–9]. When Bowen [10] introduced the family life theory, he surmised that families are complex units that are closely intertwined, with each member having a large impact on others. Unsurprisingly, with more members (e.g., children) of this unit, maintaining a peaceful and healthy state may become even more challenging [11].

Several theories that motivate marital satisfaction research provide rationale to the expectation that as the family (i.e., number of children) grows, the relationship between spouses is being challenged. One of the most prominent and highly cited perspectives–social exchange theory–builds on Thibaut and Kelly’s theory of interdependence [12] and suggests that people involve in a particular relationship when this relationship provides a satisfactory costs-to-benefits ratio [13–15]. As number of offspring raises, parents invest more and more time and efforts to take care of the children. By doing so, remaining resources to being taken care of by their partner may remain scarce. Having more children may be, therefore, seen as a binding factor and a barrier to leave the marriage, what forms a stable–but not necessarily satisfied–dyad [16].

Not only is raising children time-consuming and tiring, it is also related to a frequent exposure to stressors. Thus, another essential theory on marital satisfaction is the crisis theory [17], which focuses on crisis management and capitalizes on stressful events, coping resources, definitions of events that modify stressors’ impact, and response to the crisis. With a growing number of children, a number of stressors grows alike. The stressors may outweigh the resources that couple posits at some point. Even if partners are fulfilled as parents, their relational wellbeing may be threatened due to parental distress [18].

Indeed, some research found a negative relation between these variables: parenthood was associated with decreased marriage quality [19], increased marital conflict [20], more severe symptoms of depression [21], and decreased marital satisfaction [22]–especially when pregnancy was unplanned [5]. Other studies suggested a positive or no relation between the number of children and marital satisfaction [2, 4, 5, 23]. For instance, Yu et al. [24] analyzed an impressively large dataset of 72,668 adults and found that being a parent was positively linked to increased self-reported well-being. Furthermore, Kohler et al. [25] provided evidence that a first-born child increases overall happiness both among men and (even more) among women, but subsequent children do not influence happiness ratings, or may even decrease levels of happiness among mothers.

It seemed that the meta-analysis conducted by Twenge and colleagues [26] could bring final conclusions: the authors suggested that an increased number of children in a family decreases reports of marital satisfaction. But more recent studies again showed different directions of this link, especially in the non-Western countries [9, 27–29]. The main limitation of the previous studies is that they were conducted almost exclusively in Westernized samples (e.g., [26, 30]), and, therefore, results cannot be generalized to other societies. The goal of the present analysis is to determine the relationship between marital satisfaction and the number of children, as well as its moderators (previously reported as relevant).

One of the predictors of marital satisfaction, long since identified in the literature, is sex, with men being typically more satisfied than women [31–33]. Also in the parenting context, the relationship between the transition to parenthood and the decline of marital satisfaction is stronger for women than men [6, 26, 34]. According to equity theory (akin to social exchange theory), participation in inequitable relationship is a predictor of distress [35]. Both over-benefitted and under-benefitted partners may be dissatisfied in an imbalanced relationship [36]. At the same time, from the perspective of social role theory, the importance of different expectations to fulfill home-related responsibilities varies across both sexes [37]. Men are socially expected to provide for their families outside of the home, while women are culturally encouraged to stay within the home realms, fulfilling tasks related to housekeeping and childrearing. Different social roles and norms do not imply that both sexes are equally content with the labor division. In some cases, spouses may experience asymmetry between their commitment and investment in the relationship and rearing children. In fact, Gjerdingen and Chaloner [34] connected new mothers’ spousal dissatisfaction to insufficient men’s contribution to growing household duties, and Dew and Wilcox [6] further attributed the effect to reductions in wives’ quality time spent with their husbands after becoming a parent. Taking less care for spousal relationship or having reduced quality time should negatively affect spousal relationship among women more than among men, and that dissatisfaction can increase with commitment and time devoted to subsequent children. In addition, as women who give birth to more children appear less attractive to men than those with lower parity [38], such negative feelings of being less attractive may further translate into lower marital satisfaction.

Economic factors are additional key variables in predicting marital satisfaction [39–41]. Low-income or material hardship is associated with a serious threat to marital quality and stability [42]. Many researchers took under consideration the influence of a spouse education level on marital satisfaction [43, 44], but we found scarce data and equivocal results regarding links between the education level and a transition to parenthood [45]. So far, it was shown that highly educated men benefit more from fatherhood in terms of happiness compared to their less educated peers; no such link was found among women [46]. On the other hand, Nomaguchi and Brown [47] provided evidence for a different relation: more educated women that had fewer children perceived less benefits from parenting. In this context, education may be considered not only an obligation to invest in one’s own needs, career or childrearing, but also as a supportive resource, which gives tools or opens new possibilities. Moreover, even though previous studies provided evidence that marital satisfaction may not be related to religious affiliation, i.e., that Christians, Muslims, and atheists report the same levels of marital satisfaction [48], some researchers hypothesized that it may be rather the intensity of religiousness that affects the spouse satisfaction and parenthood [6].

Importantly, all those variables were investigated almost exclusively in Western countries. Meanwhile, social norms build, inter alia, a wide context of specific rules about family, or parenting and marriage relationship, which are customized to values they promote [9, 28]. The criteria for a satisfying marriage may vary and rely on a larger cultural context, for instance, whether the society promotes more collectivistic or individualistic values [49]. If individuals profess collectivistic norms, they are more concentrated on mutual help, loyalty, and cooperation in intra-group relationships, and because of preferring more group than individual needs, as well as getting help from relatives with children rearing, this way of life might increase their marital satisfaction [9]. As most Western countries are extremely individualistic, we aimed to re-examine the link between marital satisfaction and the number of children also in non-Western and collectivistic cultures.

Considering the above, we hypothesized that less satisfied with their marriages may be parents with more children and facing more material hardship, mothers, less religious, and those with less individualistic values. We had no prior hypotheses regarding links between the marital satisfaction, children and education. We utilize a large, cross-national dataset in the hope to obtain more generalizable results than previous studies did and provide empirical test to mechanisms suggested by classical theories informing marital satisfaction studies.

## Method

The study protocol received ethical approval from the Institutional Ethics Committee at the Institute of Psychology at the University of Wroclaw. All participants gave written informed consent in accordance with the Declaration of Helsinki.

### Participants

Data for the study was obtained from the published dataset [50], which include 7178 married individuals from 33 countries and territories: Brazil, Bulgaria, Canada, China, Croatia, Estonia, Germany, Ghana, Greece, Hong Kong, Hungary, India, Indonesia, Iran, Italy, Kazakhstan, Kenya, Malaysia, Mexico, Nigeria, Pakistan, Poland, Portugal, Romania, Russia, Saudi Arabia, Slovakia, South Korea, Spain, Switzerland, Turkey, United Kingdom, and Uganda. The data was collected in 2012 and 2013 and was part of a broad cross-cultural research project, which investigated, inter alia, romantic relationships [48, 51–54], behaviors [55], and their motives [56] across numerous countries and territories. All samples were convenience samples (e.g., students, acquaintances of the researchers, participants of vocational courses, inhabitants of home towns of the researchers etc.). With an exception of two countries (Switzerland and Bulgaria), all participants completed the paper-and-pencil questionnaires. On average, the participants were 40.7 years old (*SD* = 11.5, range = 17–88), had been married for 14.8 years (*SD* = 11.6, range = 0.8–70), had 1.7 children (*SD* = 1.3, range = 0–12), were moderately religious (median = 4, on a 1–7 point scale, with higher values representing being more religious), were more individualistic (median = 5.5, on a 1–7 point scale, with higher values representing being more individualistic), and reported their economic status as being average compared with others (median = 3, on a 1–5 point scale, with higher values representing being more wealthy than others). More than half participants attained bachelor or master’s degree (52.6%). More detailed description of data collection procedures and descriptive statistics are available in Sorokowski et al. [50].

### Measures

From the dataset (which can be found under the link: https://figshare.com/s/172d436cf55e289a85d8), we selected and analyzed following variables: marital satisfaction, the number of children, and sociodemographic variables (including sex, religiosity, economic status, education, and level of individualistic values). The data from the Marriage and Relationships Questionnaire (MRQ), developed by Russell and Wells [57], was adopted as an indicator of the participants marital satisfaction level. The authors of dataset [50] used the 9-item version of the MRQ (“Love Scale”), psychometrically appropriate for international use [58–60]. Sample questions from this scale included: “*Do you enjoy your husband’s/wife’s company*?"; "*Do you enjoy doing things together*?". Participants answered these questions on the five-point scale, which ranged from “*yes* (1)” to “*no* (5)”; answers were recoded so that higher values indicated higher marital satisfaction.

Religiosity was measured using a single item (“*Are you religious*?”), and responses ranged from 1 (*not at all*) to 7 (*extremely religious*). Economic status was measured by asking participants to rate their material situation on a five-point scale, where (1) meant–“*much better than average in my country”*, and (5)–“*much worse than average in my country”*. Answers were recoded so that higher value indicated better material situation. Individualism was measured by a scale taken from GLOBE survey [61], but we included only items regarding familiar individualism (seven-point scale–from 1, “*strongly agree*”, to 7, “*strongly disagree*”). A sample question from this scale included: “*I think children can live at home with their parents until they get married*” or “*I think parents should take pride on the individual accomplishments of their children*”. Higher values indicated higher individualism. For more information see Sorokowski et al. [50].

## Results

**Fig 1:**
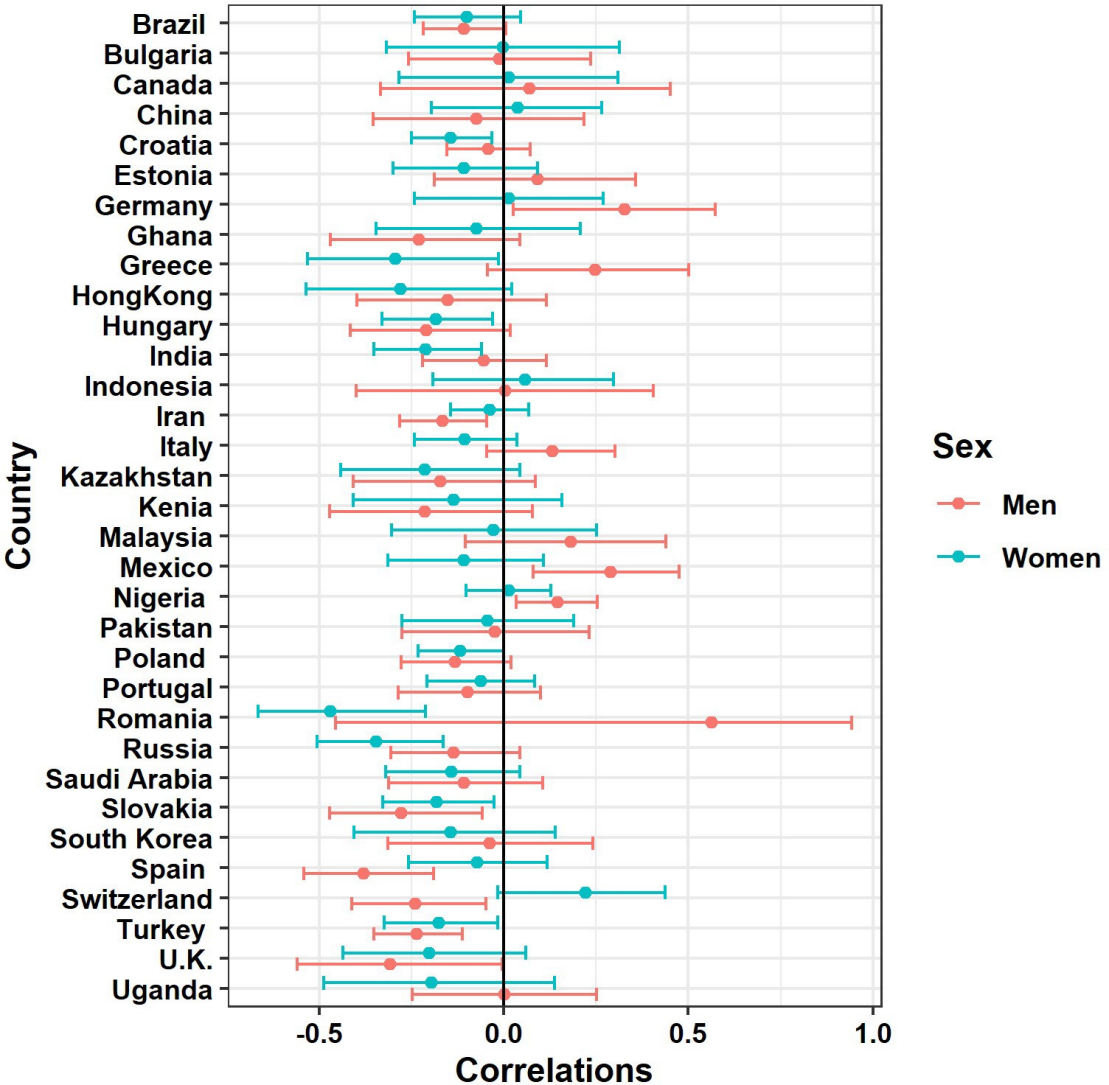
Relationships (Pearson *r*) and 95% confidence intervals between the number of children and marital satisfaction with respect to sex and countries.

Detailed information on the mean number of children across countries is presented in Table 1. For a graphical representation, see Fig 1.

**Table 1:** Characteristics of participants’ number of children across the countries.

Country	Mean number of children (SD)	Number of children (range)
Brazil	1.08 (1.02)	0–6
Bulgaria	1.06 (0.46)	0–2
Canada	0.83 (0.99)	0–4
China	0.98 (0.49)	0–3
Croatia	1.72 (1.06)	0–8
Estonia	1.93 (1.09)	0–5
Germany	1.69 (1.01)	0–5
Ghana	2.47 (1.55)	0–6
Greece	1.54 (1.04)	0–4
Hongkong	1.49 (1.09)	0–6
Hungary	1.61 (0.99)	0–7
India	0.99 (0.76)	0–3
Indonesia	2.02 (1.05)	0–5
Iran	2.02 (1.55)	0–12
Italy	1.67 (0.88)	0–4
Kazakhstan	1.87 (0.62)	0–3
Kenia	1.82 (1.24)	0–7
Malaysia	2.89 (1.98)	0–8
Mexico	1.62 (1.14)	0–6
Nigeria	2.54 (1.77)	0–10
Pakistan	1.84 (1.38)	0–6
Poland	1.82 (1.19)	0–8
Portugal	1.64 (0.83)	0–5
Romania	0.94 (0.77)	0–2
Russia	1.02 (0.84)	0–5
Saudi Arabia	2.81 (1.72)	0–8
Slovakia	1.80 (0.99)	0–8
South Korea	1.65 (0.76)	0–3
Spain	1.75 (0.88)	0–5
Switzerland	2.04 (1.28)	0–8
Turkey	1.75 (1.16)	0–7
U.K.	1.72 (1.36)	0–6
Uganda	2.73 (2.12)	0–11

In order to assess whether the number of children predicts marital satisfaction, we ran a series of multilevel linear models. All predictors were grand-mean centered. As the first step, we ran a baseline model, ignoring the data hierarchical structure with number of children, sex, age, marriage duration, religiosity, education, material situation and individualism as predictors of marital satisfaction. In the next step (model 2), we clustered data in countries and included random intercept. In the third model, we introduced interaction terms (number of children * religiosity / education / sex / economic status / individual level individualistic values). In the final model, we included random intercept and random slopes, hence, we allowed both intercept and slopes to vary across countries. We compared the models using -2 log likelihood (-2LL) with lower values indicating better models. We estimated effects using a maximum likelihood estimator.

Each subsequent model provided better fit to the data according to -2LL; χ^2^(1) = 774.32, *p <* 0.001 between the first and the second model, *χ*^2^(2) = 23.83. *p <* 0.001 between the second and the third model and *χ*^2^(5) = 21.98, *p <* 0.001 between third and fourth model. All steps–accounting for data hierarchical structure, introducing interaction terms and allowing slopes to vary (and covary with intercepts)–changed significance of the parameter estimate for the number of children as a predictor of marital satisfaction. Results of the final model are presented in Table 2. Intra-class correlation coefficient (ICC) demonstrated that 13% of the variance of marital satisfaction was associated with country level and 87% was assigned to individual level. In the final model, number of children predicted marital satisfaction (*β* = -0.030, *p* = 0.048), but the effect was relatively small. Age negatively predicted marital satisfaction (*β* = -0.010, *p* < 0.001). Sex was also found a significant predictor (*β* = -0.010, *p* < 0.001) with men being more satisfied than women. Remaining predictors: education (*β* = 0.057, *p* < 0.001), religiosity (*β* = 0.048, *p* < 0.001), material situation (*β* = 0.152, *p* < .001) and individualism (*β* = 0.064, *p* < 0.001) were positively related to marital satisfaction. Marriage duration was not a significant predictor (*β* = -0.004, *p* = 0.099). The interactions between the number of children and religiosity (*β* = 0.012, *p* = 0.020) / sex (*β* = -0.040, *p* = 0.019) / education (*β* = -0.025, *p* = 0.004) were found to be significant, unlike interactions between the number of children and material situation and the individual level of individualism (*p* = .634 and *p* = 0.105, respectively).

**Table 2:** Results of the multilevel linear model regressing marital satisfaction on number of children, sex, age, marriage duration, religiosity, education, material situation, individualism, and number of children x religiosity/education/sex/material situation/individualism.

	Marital Satisfaction				
Fixed Effects	*β*	*CI (95%)*	*SE*	*Statistic*	*p*
Intercept	0.053	-0.084–0.189	0.070	0.760	0.448
Sex (-.5 = Men, .5 = Women)	-0.099	-0.143 – -0.056	0.022	-4.503	**<0.001**
Number of Children	-0.030	-0.060 – -0.000	0.015	-1.974	**0.048**
Age	-0.010	-0.014 – -0.006	0.002	-4.766	**<0.001**
Education	0.057	0.032 – 0.082	0.013	4.449	**<0.001**
Material Status	0.152	0.123 – 0.180	0.014	10.515	**<0.001**
Religiosity	0.048	0.034 – 0.062	0.007	6.960	**<0.001**
Individualism	0.064	0.043 – 0.085	0.011	6.025	**<0.001**
Marriage Duration	-0.004	-0.008 – 0.001	0.002	-1.648	0.099
Sex x Children	-0.040	-0.073 – -0.007	0.017	-2.354	**0.019**
Children x Education	-0.025	-0.042 – -0.008	0.009	-2.886	**0.004**
Children x Material Status	0.005	-0.015 – 0.024	0.010	0.477	0.634
Children x Religiosity	0.012	0.002 – 0.021	0.005	2.331	**0.020**
Children x Individualism	0.012	-0.003 – 0.027	0.007	1.620	0.105
**Random Effects**					
σ^2^	0.82				
τ_00 Country_	0.12				
τ_11 Children_	0.00				
ρ_01 Country_	0.55				
ICC	0.13				
N _Country_	33				
Observations	7178				
Marginal R^2^ / Conditional R^2^	0.076 / 0.199				

Significant results are bolded.

**Fig 2:**
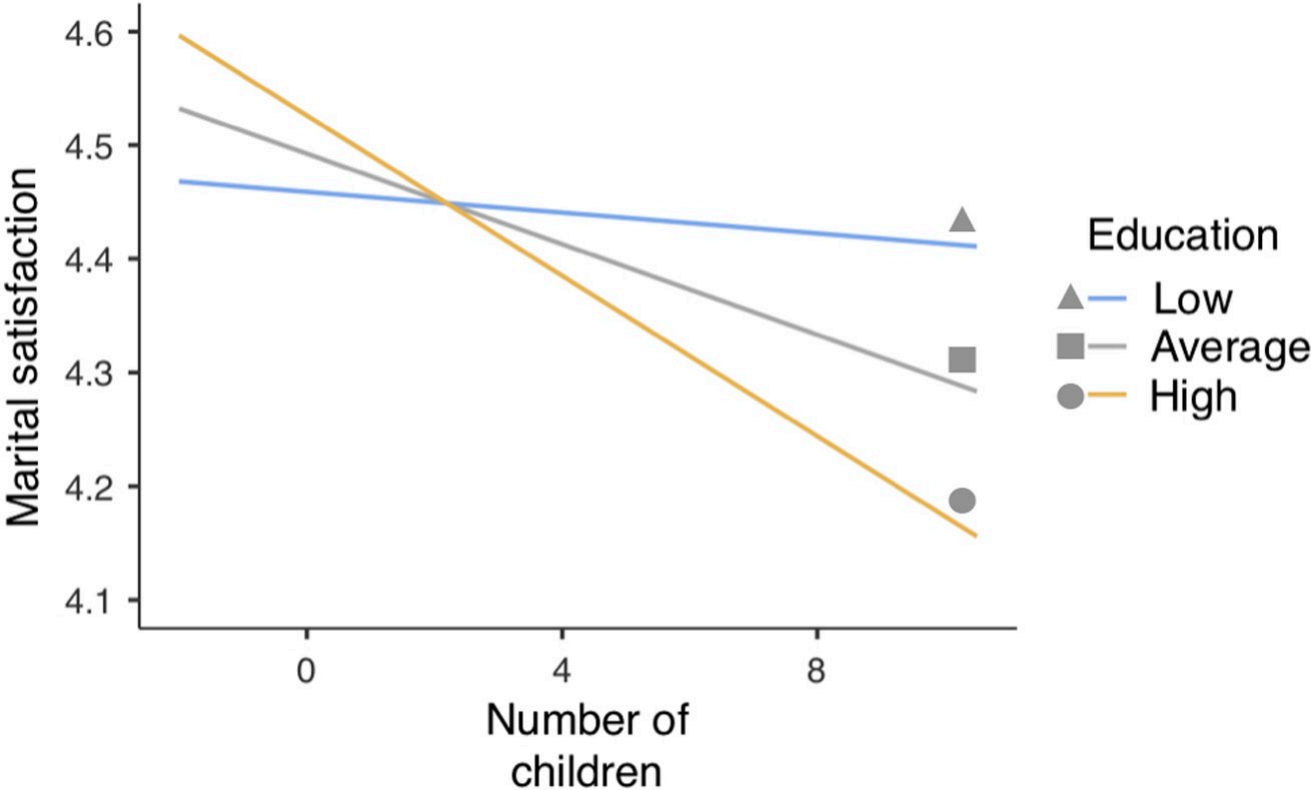
Interaction of the number of children and education in relation to marital satisfaction (MRQ).

Next, in order to break down the interactions, we ran a series of simple slope analyses to examine the relationship between the number of children and marital satisfaction at mean value and +/- 1 *SD* of moderators, controlling for other variables. The results showed that there was no effect of the low (-1 *SD*) level of education on the number of children and marital satisfaction (*β* = -0.007, *t* = -0.401, *p* = 0.690), yet, there were significant effects of the high and mean levels of education (*β* = -0.030, *t* = -1.974, *p* = 0.055, *β* = -0.054, *t* = -3.012, *p* = 0.003, respectively). These results suggest that for a group of high and mean education, the higher the number of children, the lower the marital satisfaction (Fig 2).

**Fig 3:**
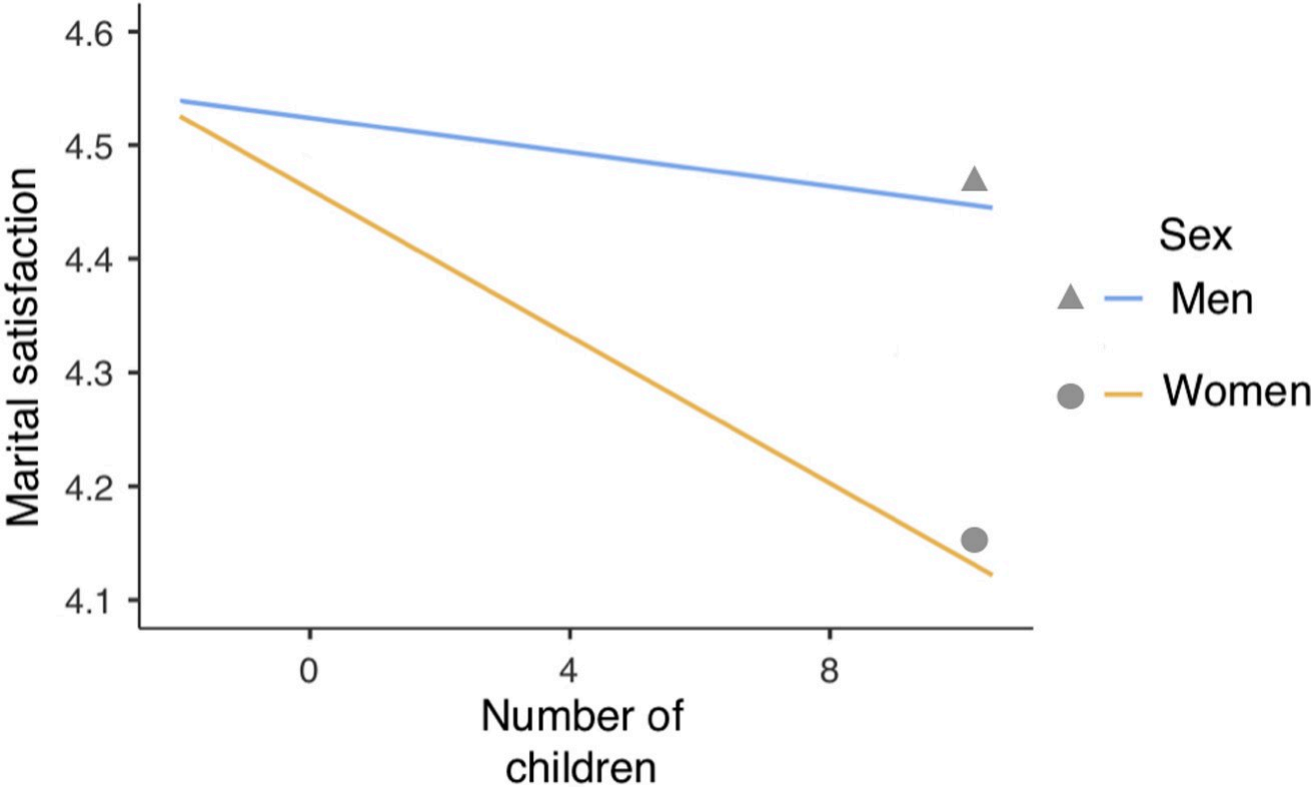
Interaction of the number of children and religiosity in relation to marital satisfaction (MRQ).

**Fig 4:**
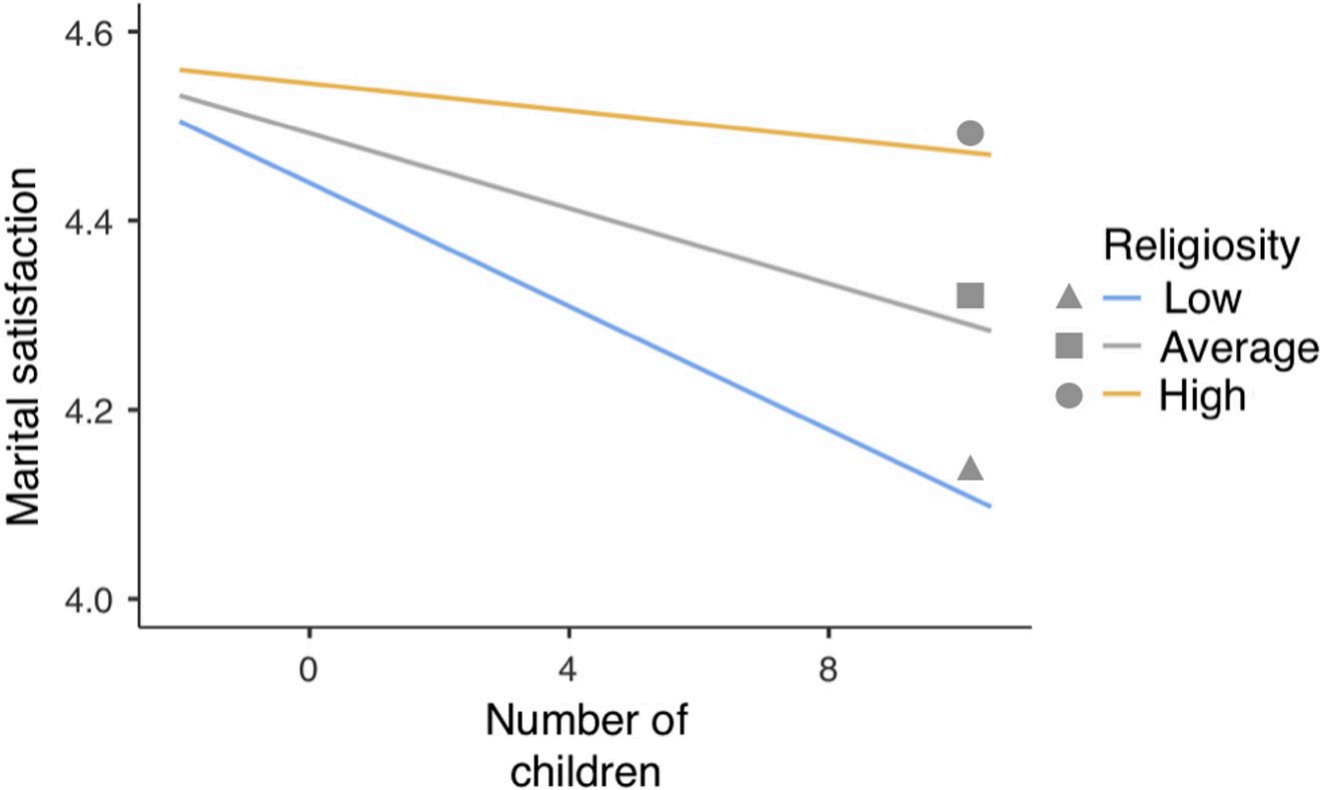
Interaction of the number of children and sex in relation to marital satisfaction (MRQ).

Breaking down the interaction between the number of children and religiosity revealed that in participants at high (+1 *SD*) level of religiosity the number of children did not significantly predict marital satisfaction (*β* = -0.010, *t* = -0.562 *p* = 0.576), however this relationship was almost significant and negative in participants at mean level of religiosity (*β* = -0.030, *t* = -1.974, *p* = 0.055), and significant at low (-1 *SD*) level of religiosity (*β* = -0.051, *t* = -2.811, *p* = 0.006) (Fig 3). It implies that among respondents with a low level of religiosity, the higher the number of children, the lower the marital satisfaction. Among women, the relationship between marital satisfaction and number of children was significant and negative (*β* = -0.050, *t* = -2.896, *p* = 0.005) but it was not significant for men (*β* = -0.011, *t* = -0.594, *p* = 0.555) (Fig 4).

The results show that the variance of both–slopes and intercepts was significant, indicating that the effect of the number of children on marital satisfaction is unstable across countries. All the analyses were performed in R (version 4.0.3) and in Jamovi (version 1.6.2).

## Discussion

Our findings are in line with other research [26, 28], which showed that the number of children can be considered as a global, negative correlate of marital satisfaction. Even though some previous studies found that being a parent (as compared to non-parents) is linked to increased overall well-being [1, 2] (and that there are pronounced, cross-cultural differences within this matter, e.g., between American and Chinese adults [62]), the current analyses seem to refute the notion that such beneficial influence of parenthood extends to marital satisfaction. Moreover, as much greater share of variance can be attributed to individuals than to countries, one can reasonably conclude that marital satisfaction depends more on the individual characteristics than on the values promoted in the country. At the same time, we found that the association between marital satisfaction and the number of children vary substantially across countries, what necessitates further investigations.

Our study provided evidence for the complexity and the influence of other variables on the link between marital satisfaction and the number of children, namely, sex, education, and religiosity. We observed that a higher number of children was associated with decreased marital satisfaction only among women. According to the social role theory [37], it is women who are culturally pressured to fulfill tasks related to childbearing and housekeeping, while men provide for their families outside of the home. In such a situation, having more children generates more home duties for mothers than fathers [6, 34]. At the same time, as caring for children and their safety is a typical female role [37], men may solely focus on having fun and playing with the offspring [2], and thus, men may experience less distress and, in turn, more positive emotions regarding their spouse. Considering the imbalance between spouses’ duties related to having more children, results of the present study are in line with the equity theory [35], which predicts that partners, who invests more in the relationship than their spouses, experience more severe distress.

In addition to the sex differences, our analyses showed the interactive effect of the number of children and the level of parental education on marital satisfaction. Previous findings suggested that higher level of parental education should facilitate family size planning and achieving a balance between familial and personal life goals by both parents [63]. However, our results advocate for the opposite–we observed that the more education parents receive, the lower levels of marital satisfaction they experienced. When higher educated parents have more children, they may encounter more difficulties in balancing various social roles. This situation may result from the limitations of time and personal resources necessary to reconcile satisfyingly fulfilling parental, partner, and professional roles at the level determined by generally available knowledge [64].

We hypothesized that material status may be interacting with the number of children and marital satisfaction. Surprisingly, we found no support for this hypothesis. Parents of more children, regardless of their material situation, reported lower levels of marital satisfaction. Two complementary mechanisms may explain these findings. First, according to the restriction of freedom model [26], parents of high material status may more severely perceive a greater restriction of their free time. Instead of pursuing desirable careers or fulfilling dreams that would otherwise be financially affordable, parents focus on their offspring (who require time and attention). Second, according to the financial cost model [26], having children entails a myriad of expenses. With more children, it is even more difficult to make ends meet. Also, economic problems may be associated with husbands’ increased hostility and decreased supportiveness, both leading to wives’ perceptions of lower marital quality [39]. On the other hand, Twenge et al. [26] showed that when a couple becomes parents, a relationship between the transition to parenthood and the decline of marriage satisfaction may be stronger for individuals of higher socioeconomic status. Thus, we conclude that when the number of children increases, neither good nor bad material situation protects spouses from experiencing decreased levels of marital satisfaction. Similarly, in case of individualism. Previous studies found that parents from Western countries, usually recognized as more individualistically oriented [49], experience a decrease of marital satisfaction upon birth of their children [26], and thus, we hypothesized that more level of individualistic values may interact with marital satisfaction and the number of children. However, we found no evidence for the influence of individualism on this relationship.

Analyzing the impact of religiosity on the number of children and marital satisfaction, we observed that religiosity may be a protective buffer against a marital satisfaction decrease in larger families. Many religious communities stress positive marital and family relations [65, 66], offer different forms of support to parents [67], and value parenting likewise bringing up children through religious teachings, ceremonies or accommodations to families with children [65, 68]. Furthermore, religious people may not consider maternity in terms of inner conflict between individual aims and parent obligations [6, 69]. On the contrary, religiosity may promote traditional roles (i.e., being a parent, a spouse), and thus, positively influence the link between parenthood and marital satisfaction [70–72].

The correlations between the number of children and marital satisfaction differed across countries (see Fig 1), being positive in few cases (only among men) and negative in others. However, these correlations were never strong. The plot suggests no emerging patterns that could condition the direction and intensity of these relationships (e.g., a positive relationship in men in Germany, Nigeria, and Mexico). However, a positive effect of individualism on marital satisfaction suggests that it remains dependent on culturally determined issues. Although individualism did not differentiate the relationship of our interest, some country-level or other culturally relevant aspects of spouses’ functioning should be tested in future studies. For example, work culture [73], country policies [74] or social equity norms shared within a society [36] may explain to a higher extent the cultural differences in the role that number of children play in marital satisfaction. Nevertheless, due to the limitations described below we want to stress that the present results should be treated with caution until future cross-cultural studies provide further support.

### Strengths and limitations

Results of the present analysis are not free of limitations. Most importantly, the statistical significance of the observed relationship between marital satisfaction and the number of children was very close to the conventional threshold of 0.05. We cannot exclude the scenario in which the significance of this predictor might have been a result of a large sample size, what required caution in drawing any general conclusions. Furthermore, the data samples are not fully representative for the whole world’s population, as the majority of participants inhabited more urbanized regions. We were also unable to analyze interdependent marriage dyads or non-married, cohabitating couples. Moreover, religiosity appeared to be a moderator of the link between the number of children and marital satisfaction, but, unfortunately, it was assessed only by a single question in the survey (“*Are you religious*?”), which makes further interpretations difficult. The partial, declarative knowledge of participants economic status also limits our conclusions. It would be insightful if future studies focused on the age of the children, as it may also affect the relationship between the family size and marital satisfaction. Finally, our study did not focus on very complex relationships between our variables of interest (i.e., three-way-interactions). We suggest that building upon sound theoretical backgrounds, future studies could form more detailed hypotheses on the interplay between several predictors of marital satisfaction and their temporal dynamics.

On the other hand, in the present analysis we used a large-scale sample database from different regions of the world. All participants answered the same questionnaires, which tried to capture numerous important variables, previously shown to correlate with marital satisfaction. The data was collected in the same period of time and originated in different regions of the world. The main contribution of the present research is extending our knowledge on the relationship between marital satisfaction and the number of children and variables that are frequently hypothesized to influence this relationship (i.e., sex, religiosity, age, education, level of individualism, material situation, and marriage duration) in several, non-Western countries and territories. Such insight may be especially important when considering the importance of marital satisfaction on health and well-being both of spouses [75] and their children [76].

## Supporting information

S1 File (DOCX)
