# Supplementary material for: When and how does the number of children affect marital satisfaction? An international survey
Source: PLoS One. 2021 Apr 22;16(4):e0249516. doi: 10.1371/journal.pone.0249516 (PMC8062063; doi:10.1371/journal.pone.0249516)
Supplement: S1 File — (DOCX) [file pone.0249516.s001.docx]

| S1 Table. Marital satisfaction across different levels of number of children | | | | | | | | | | | | |
| --- | --- | --- | --- | --- | --- | --- | --- | --- | --- | --- | --- | --- |
|  |  |  | |  |  |  |  |  |  |  |  |  |
|  | **Number of children** | | | | **N** | | **Mean** | | **SD** | | **SE** | |
|  |  | | 0 |  | 1060 |  | 4.65 |  | 0.514 |  | 0.016 |  |
|  |  | | 1 |  | 2074 |  | 4.49 |  | 0.638 |  | 0.014 |  |
|  |  | | 2 |  | 2579 |  | 4.46 |  | 0.640 |  | 0.013 |  |
|  |  | | 3 |  | 888 |  | 4.45 |  | 0.663 |  | 0.022 |  |
|  |  | | 4 |  | 323 |  | 4.46 |  | 0.648 |  | 0.036 |  |
|  |  | | 5 |  | 254 |  | 4.37 |  | 0.730 |  | 0.046 |  |
|  | | | | | | | | | | | | |

| S2 Table. Results of the ANOVA analysis with the marital satisfaction as a dependent variable and categorized variable children as a fixed factor. | | | | | | | | | | | | | |
| --- | --- | --- | --- | --- | --- | --- | --- | --- | --- | --- | --- | --- | --- |
|  |  |  |  |  |  |  |  |  |  |  |  |  |  |
|  | | **Sum of Squares** | | **df** | | **Mean Square** | | **F** | | **p** | | **η²** | |
| Overall model |  | 87.883 |  | 5 |  | 17.577 |  | 17.782 |  | < .001 |  |  |  |
| Children ^a^ |  | 87.883 |  | 5 |  | 17.577 |  | 17.782 |  | < .001 |  | 0.012 |  |
| Residuals |  | 7089.117 |  | 7172 |  | 0.988 |  |  |  |  |  |  |  |
| Note. ^a^ Children–a categorized variable with 6 levels (i.e., 0, 1, 2, 3, 4, 5 or more children). | | | | | | | | | | | | | |

| S3 Table. Tuckey post-hoc tests for the ANOVA analysis with the marital satisfaction as a dependent variable and categorized variable children as a fixed factor.. | | | | | | | | | | | | | | | |
| --- | --- | --- | --- | --- | --- | --- | --- | --- | --- | --- | --- | --- | --- | --- | --- |
|  |  |  |  |  |  |  |  |  |  |  |  |  |  |  |  |
|  | |  | | **0** | | **1** | | **2** | | **3** | | **4** | | **5** | |
| 0 |  | Mean difference |  | — |  | 0.163 |  | 0.1896 |  | 0.1998 |  | 0.195 |  | 0.285 |  |
|  |  | p-value |  | — |  | < .001 |  | < .001 |  | < .001 |  | < .001 |  | < .001 |  |
| 1 |  | Mean difference |  |  |  | — |  | 0.0269 |  | 0.037 |  | 0.032 |  | 0.123 |  |
|  |  | p-value |  |  |  | — |  | 0.697 |  | 0.684 |  | 0.956 |  | 0.039 |  |
| 2 |  | Mean difference |  |  |  |  |  | — |  | 0.010 |  | 0.006 |  | 0.096 |  |
|  |  | p-value |  |  |  |  |  | — |  | 0.998 |  | 1.000 |  | 0.188 |  |
| 3 |  | Mean difference |  |  |  |  |  |  |  | — |  | -0.005 |  | 0.086 |  |
|  |  | p-value |  |  |  |  |  |  |  | — |  | 1.000 |  | 0.395 |  |
| 4 |  | Mean difference |  |  |  |  |  |  |  |  |  | — |  | 0.09 |  |
|  |  | p-value |  |  |  |  |  |  |  |  |  | — |  | 0.524 |  |
| 5 |  | Mean difference |  |  |  |  |  |  |  |  |  |  |  | — |  |
|  |  | p-value |  |  |  |  |  |  |  |  |  |  |  | — |  |
|  | | | | | | | | | | | | | | | |

| S4 Table. Results of the ANCOVA analysis with the marital satisfaction as a dependent variable and added remaining variables of interest as independent variables. | | | | | | | | | | | | | |
| --- | --- | --- | --- | --- | --- | --- | --- | --- | --- | --- | --- | --- | --- |
|  |  |  |  |  |  |  |  |  |  |  |  |  |  |
|  | | **Sum of Squares** | | **df** | | **Mean Square** | | **F** | | **p** | | **η²** | |
| Overall model |  | 209.714 |  | 42 |  | 4.993 |  | 12.889 |  | < .001 |  |  |  |
| Children ^a^ |  | 14.253 |  | 5 |  | 2.851 |  | 3.049 |  | 0.009 |  | 0.002 |  |
| Sex (-0.5-Men, 0.5-Women) |  | 7.057 |  | 1 |  | 7.057 |  | 7.548 |  | 0.006 |  | 0.001 |  |
| Age |  | 2.235 |  | 1 |  | 2.235 |  | 2.390 |  | 0.122 |  | 0.000 |  |
| Marriage Duration |  | 12.815 |  | 1 |  | 12.815 |  | 13.707 |  | < .001 |  | 0.002 |  |
| Education |  | 7.079 |  | 1 |  | 7.079 |  | 7.571 |  | 0.006 |  | 0.001 |  |
| Material Status |  | 100.137 |  | 1 |  | 100.137 |  | 107.104 |  | < .001 |  | 0.015 |  |
| Religiosity |  | 0.522 |  | 1 |  | 0.522 |  | 0.559 |  | 0.455 |  | 0.000 |  |
| Individualism |  | 3.880 |  | 1 |  | 3.880 |  | 4.150 |  | 0.042 |  | 0.001 |  |
| Children x Sex |  | 10.752 |  | 5 |  | 2.150 |  | 2.300 |  | 0.042 |  | 0.002 |  |
| Children x Marriage Duration |  | 7.580 |  | 5 |  | 1.516 |  | 1.622 |  | 0.151 |  | 0.001 |  |
| Children x Education |  | 23.387 |  | 5 |  | 4.677 |  | 5.003 |  | < .001 |  | 0.003 |  |
| Children x Material Status |  | 7.807 |  | 5 |  | 1.561 |  | 1.670 |  | 0.138 |  | 0.001 |  |
| Children x Religiosity |  | 3.338 |  | 5 |  | 0.668 |  | 0.714 |  | 0.613 |  | 0.000 |  |
| Children x Individualism |  | 8.872 |  | 5 |  | 1.774 |  | 1.898 |  | 0.091 |  | 0.001 |  |
| Residuals |  | 6670.881 |  | 7135 |  | 0.935 |  |  |  |  |  |  |  |
| Note. ^a^ Children–a categorized variable with 6 levels (i.e., 0, 1, 2, 3, 4, 5 or more children). | | | | | | | | | | | | | |
